# Impaired Kynurenine Pathway in Inflammatory Bowel Disease

**DOI:** 10.3390/jcm13206147

**Published:** 2024-10-15

**Authors:** Esra Paydaş Hataysal, Muslu Kazım Körez, Eray Metin Guler, Hakan Vatansev, Kubra Bozalı, Metin Basaranoglu, Husamettin Vatansev

**Affiliations:** 1 Department of Biochemistry, Göztepe Prof. Dr. Süleyman Yalçın City Hospital, 34722 Istanbul, Türkiye; 2 Department of Biostatistics, Faculty of Medicine, Selcuk University, 42250 Konya, Türkiye; 3 Department of Biochemistry, Hamidiye Faculty of Medicine, University of Health Sciences, 34480 Istanbul, Türkiye; 4 Department of Food Processing, Meram Vocational School, Necmettin Erbakan University, 42092 Konya, Türkiye; 5 Department of Gastroenterology, Faculty of Medicine, Bezmialem University, 34093 Istanbul, Türkiye; 6 Department of Biochemistry, Faculty of Medicine, Selcuk University, 42250 Konya, Türkiye

**Keywords:** inflammatory bowel diseases, Crohn’s disease, ulcerative colitis, tryptophan, kynurenine pathway

## Abstract

**Background/Objectives**: Inflammatory bowel diseases primarily encompass Crohn’s disease and ulcerative colitis. Insufficient levels of tryptophan cause an imbalance in the gut microbiota, leading to inflammation in the gastrointestinal tract. The main catabolic pathway of tryptophan is the kynurenine pathway. Our study aims to evaluate serum tryptophan, the kynurenine pathway, and oxidative stress parameters, including total oxidant status and total antioxidant capacity, in patients with Crohn’s disease and ulcerative colitis. **Methods**: The study included 80 follow-up patients in remission diagnosed with Crohn’s disease and ulcerative colitis who attended the Gastroenterology Outpatient Clinic, as well as 78 healthy controls. Serum tryptophan, kynurenine, 3-hydroxykynurenine, 3-hydroxyanthranilic acid, and kynurenic acid levels were measured with liquid chromatography and tandem mass spectrometry (LC-MS/MS). All statistical analysis was performed using R version 4.2.1. Statistical Language. **Results**: Serum tryptophan, 3-hydroxyanthranilic acid, and total antioxidant capacity were lower in patients with ulcerative colitis and Crohn’s disease compared to those in the control group. The serum total oxidant status in the control group was significantly lower than in patients with Crohn’s disease and ulcerative colitis. **Conclusions**: The results of our research indicate that tryptophan and kynurenine pathway metabolites could potentially contribute to the pathogenesis of inflammatory bowel diseases.

## 1. Introduction

The inflammatory bowel diseases (IBDs) which constitute the most significant public health concern in developed countries are Crohn’s disease (CD) and ulcerative colitis (UC) [1]. Although the precise etiology of IBD is not well known, the specific mechanisms that result in the development of IBD are associated with improper immune system reactions, genetic predispositions, changes in the microbiota of the intestinal gut, and dietary factors [2,3,4,5]. While these conditions are frequently characterized by clinical presentations including diarrhea, abdominal pain, mucopurulent bloody stool, weight loss, and various associated symptoms, malnutrition and intestinal perforation may occur in more severe cases [6]. The global prevalence of IBD exceeds 0.3%, with the highest reported rates observed in Europe and North America [7]. It constitutes a contemporary public health challenge owing to its considerable influence on quality of life and its association with the development of colorectal cancer [8]. Hence, it is crucial to thoroughly examine the relevant pathogenesis and biochemical mechanisms in order to gain a comprehensive understanding of IBD.

In IBD, chronic inflammation and mucosal damage result in an excessive immune response and impaired tissue perfusion, leading to the overproduction of reactive oxygen and nitrogen species (ROS/RNS) [9,10]. The production of ROS by inflamed bowel tissue and inflammatory cells leads to an elevation in oxidative stress, thereby contributing to the development and progression of chronic bowel inflammation [11]. Due to the impracticality of measuring various antioxidant or oxidant molecules separately, as well as the complexity of the techniques required and the potential additive nature of their effects, total oxidant status (TOS) and total antioxidant capacity (TAC) have been introduced to assess the overall oxidant and antioxidant status [12].

Tryptophan (TRP), an essential amino acid, is a critical component of the human diet, contributing significantly to inflammatory reactions and intestinal homeostasis [6]. Insufficient levels of TRP cause an imbalance in the gut microbiota, leading to inflammation in the gastrointestinal tract and potentially throughout the body [13]. The gut microbiota plays a crucial role in regulating tryptophan metabolism via the kynurenine and serotonin pathways by producing microbial enzymes and generating metabolites [14]. Given that various factors, such as diet, antibiotics, and probiotics, can influence gut microbiota composition and metabolism, modulating TRP levels through targeted manipulation of the gut microbiota may offer a potential therapeutic strategy for numerous diseases [15]. *Hericium erinaceus*, a Chinese medicinal and edible fungus, has been shown in experimental studies to reduce proinflammatory cytokines in IBD [16], as well as strengthening the intestinal barrier through modulation of intestinal microbiota, and promoting the tryptophan metabolic pathway by increasing the abundance of Lactobacillus in the intestine [17].

**Figure 1:**
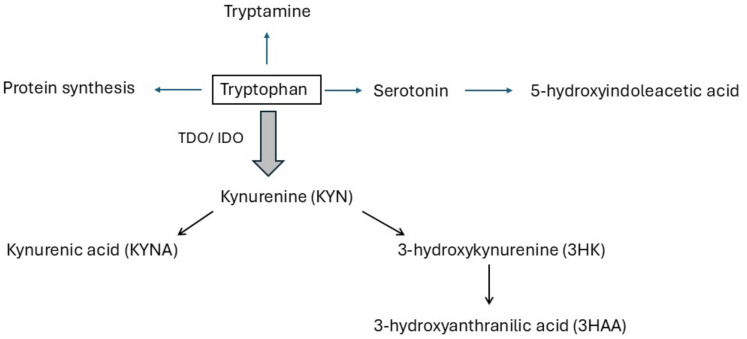
A simple diagram of the kynurenine pathway. Indoleamine 2,3-dioxygenase: IDO, tryptophan 2,3-dioxygenase: TDO.

Tryptophan undergoes metabolism through three primary biological pathways, namely the serotonin, indole-3-pyruvate and kynurenine pathways. In humans, the main catabolic route of TRP, which plays a crucial role in modulating intestinal inflammation and thereby potentially impacting the development of IBD, is the kynurenine pathway (KP), which is significant as it leads to the production of various end-product metabolites with potent biological effects, accounting for at least 90% of total TRP degradation. TRP undergoes its main metabolic transformation through the KP, yielding biologically active compounds, including kynurenine (KYN), 3-hydroxykynurenine (3HK), 3-hydroxyanthranilic acid (3HAA), and kynurenic acid (KYNA) (Figure 1). In the intricate process of metabolism, TRP undergoes degradation primarily through the actions of enzymes known as indoleamine 2,3-dioxygenase (IDO-1/IDO-2) and tryptophan 2,3-dioxygenase 2 (TDO), which catalyze the conversion of TRP to KYN. TDO regulates the fundamental breakdown of tryptophan through the KP, which primarily manages basal metabolic processes, whereas IDO predominantly governs tryptophan metabolism, particularly during immune activation in inflammatory states [18]. The stimulation of the IDO-1 enzyme by proinflammatory cytokines such as IFN-γ and TNF-α, which are produced during the immune response, indicates that the KP has significant involvement in modulating the immune system, inflammatory response, and pathogenesis of autoimmune diseases.

Recent studies have pointed towards decreased serum levels of the aromatic amino acid TRP in a number of chronic inflammatory diseases [19], with a particular emphasis on IBD [20,21,22,23]. The aim of our study was to evaluate oxidative stress parameters and serum TRP and KP through the measurement of TRP levels, along with the levels of KYN, 3HK, 3HAA, and KYNA in patients with UC and CD.

## 2. Materials and Methods

### 2.1. Study Population

A total of 80 patients with IBD (39 UC, 41 CD) in clinical remission, who were followed up at the Gastroenterology Clinic of Bezmialem University Faculty of Medicine Hospital between January 2023 and December 2023 and had been diagnosed with IBD on the basis of endoscopic, clinical, histopathological, and radiological assessments, were included in the study, along with 78 healthy controls. The control group was composed of apparently healthy participants who applied to the hospital for a check-up, who did not have any symptoms that could be confused with IBD or a family history of IBD, and who had similar demographic characteristics to the IBD group with no known chronic disease and medication use. Patients with any other chronic diseases, including cardiovascular, liver, renal diseases, active infections, diabetes, pregnancy, unusual examination findings, individuals with missing data and a history of previous malignancy, those under 18 years of age, and those with a C-reactive protein (CRP) value > 5 mg/L, were not included in the study. Clinical remission was defined as a Crohn’s Disease Activity Index (CDAI) of less than 150 for CD [24] and a total Mayo score of less than 3 for UC.

All samples were placed into BD Vacutainer SST II Advance serum gel separator tubes and BD Vacutainer K2EDTA tubes (Becton Dickinson, Franklin Lakes, NJ, USA). After centrifugation at 2000× *g* for 10 min, the serum samples were stored at 80 °C until analysis.

This study was approved by the Ethics Committee University of the Health Science, Hamidiye Faculty of Medicine (Decision Number: 27/5, Date: 16 December 2022). Informed consent was obtained from all participants, and the study was conducted in accordance with the principles outlined in the Declaration of Helsinki.

### 2.2. Laboratory Analysis

Serum glucose, creatinine, alanine aminotransferase (ALT), and aspartate aminotransferase (AST) were assessed using the Abbott Architect ci16200 (Abbott Laboratories Ltd., Abbott Park, Chicago, IL, USA) autoanalyzer, whereas a complete blood count including the white blood cell count (WBC), red blood cell count (RBC), hematocrit (HCT), mean corpuscular volume (MCV), and platelets (PLTs) was conducted utilizing a Cell-Dyn Ruby analyzer (Abbott Dıagnostıcs, Chicago, IL, USA). Serum TAC and TOS were evaluated employing an automated measurement technique (Rel Assay Diagnostics Kit, Mega Tıp, Gaziantep, Turkey), utilizing Erel’s colorimetric method [25,26]. The oxidative stress index (OSI) was computed by dividing the TOS value by the TAC value.

The quantification of TRP, KYN, 3HK, 3HAA, and KYNA concentrations was carried out via a validated tandem mass spectrometric approach, with pretreatment steps involving protein precipitation with acetonitrile followed by sample evaporation, as previously described [27]. Analyte separation was performed using a Shimadzu HPLC system (Kyoto, Japan) coupled with a Phenomenex C18 column (50 mm × 4.6 mm, 5 μm, 100 Å). The detection of KP metabolites was conducted utilizing an ABSciex API 3200 tandem mass spectrometer (Applied Biosystems/MDS Sciex) operating in positive electrospray ionization mode.

### 2.3. Statistical Analysis

All the statistical analyses were performed with R version 4.2.1. Software (The R Foundation for Statistical Computing, Vienna, Austria; https://www.r-project.org, accessed on 11 June 2024). The normality of the data was checked via Shapiro–Wilk’s normality test and the homogeneity of the variance was assessed via Levene’s test. Numerical variables are summarized as means ± standard deviations (SDs) or medians with quartiles [1st quartile–3rd quartile], as appropriate. Categorical variables are described as counts (n) and percentages (%). Depending on the distribution of the data, ANOVA or the Kruskal–Wallis test followed by Dunn’s post hoc test with a Bonferroni correction test was applied. Spearman’s rho correlation analysis was applied to assess the relationships among laboratory findings.

To determine whether there was a statistically significant difference between the IBD and control groups in terms of tryptophan metabolites and oxidative stress parameters, it was calculated that the study should be conducted with 73 patients in each group to achieve a minimum statistical power of 85% in a two-tailed independent samples *t*-test with a 5% significance level and a medium effect size of 0.5 (Cohen’s d = 0.5).

A receiver operating characteristics (ROCs) curve analysis was used for discriminating patients with IBD from healthy controls. The optimal cut-off points were obtained by the Youden index. The areas under the curves (AUCs), sensitivity, specificity, negative predictive value (NPV), and positive predictive value (PPV) were calculated in relation to the optimal cut-off points. A *p*-value of less than 0.05 was regarded as statistically significant.

## 3. Results

Patients were broadly comparable in terms of age (*p* = 0.971) and sex distribution (*p* = 0.812) among the study groups. Additionally, no significant differences were noted between the control group and the UC and CD groups in terms of laboratory findings, including glucose, creatinine, ALT, AST, WBC, RBC, HCT, MCV, and PLT values. The demographic characteristics and laboratory findings of the study groups are summarized in Table 1.

**Figure 2:**
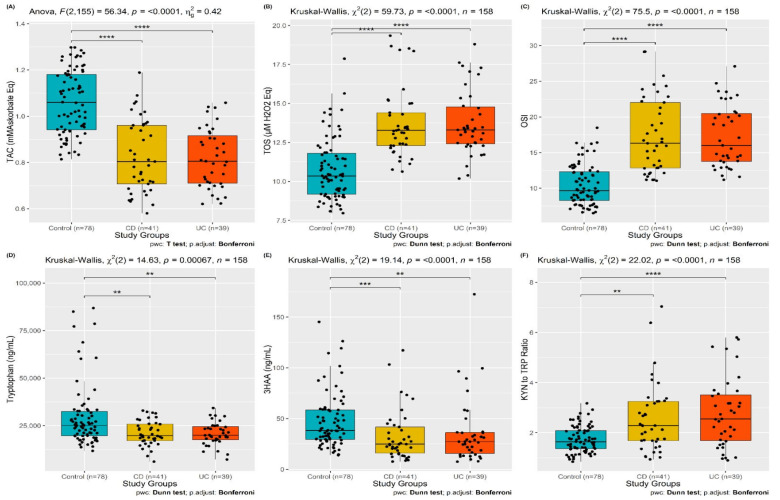
The box-plots of the serum level of TAC, TOS, OSI, Tryptophan, 3HAA and KYN/TRP ratio in patients with UC and CD and healthy control. (**A**) The serum level of TAC in patients with UC, CD and healthy controls. (**B**) The serum level of TOS in patients with UC, CD and healthy controls. (**C**) The serum level of OSI in patients with UC, CD and healthy controls. (**D**) The serum level of Tryptophan in patients with UC, CD and healthy controls. (**E**) The serum level of 3HAA in patients with UC, CD and healthy controls. (**F**) The serum level of KYN to TRP ratio in patients with UC, CD and healthy controls. (** *p* ≤ 0.01; *** *p* ≤ 0.001; **** *p* ≤ 0.0001).

In terms of TRP and its metabolites, serum TRP, 3HAA levels, KYN/TRP, and KYNA/KYN ratios were significantly different among the groups (*p* < 0.001, *p* < 0.001, *p* < 0.001 and *p* = 0.006, respectively), whereas the serum KYN, KYNA, and 3HK levels were similar across the groups (*p* = 0.265, *p* = 0.072 and *p* = 0.309, respectively). Serum TRP levels were lower in UC (19,900 [IQR, 17,550–24,600]) and CD patients (19,700 [IQR, 17,100–25,800]) than in the control group (25,100 [IQR, 19,625–32,475]) (*p* = 0.005 and *p* = 0.005, respectively). The serum 3HAA level in the control group (38.2 [IQR, 29.5–58.5]) was significantly greater than in patients with UC (27.2 [IQR, 15.5–36.2]) and CD (24.8 [IQR, 15.9–41.7]) (*p*: 0.002 and *p* < 0.001, respectively). Regarding the oxidative stress parameters, the serum TOS in the control group (10.35 [IQR, 9.18–11.81]) was significantly lower than in patients with UC (13.3 [IQR, 12.42–14.77]) and CD (13.27 [IQR, 12.29–14.41]) (*p* < 0.001 and *p* < 0.001, respectively). However, serum TAC levels were significantly elevated in the control group (1.06 ± 0.14) compared to in patients with UC (0.82 ± 0.13) and CD (0.83 ± 0.16) groups. Figure 2 shows the box-plot graph of TAC, TOS, OSI, TR, 3HAA, and KYN/TRP between the groups.

As shown in Table 2, there was a negative correlation between TOS and KYN as well as 3HK and TAC (r: −0,194, *p*: 0.015; r: −0,276, *p* < 0.001 and r: −0.666, *p* < 0.001, respectively), whereas a positive correlation was observed between TOS and 3HAA (r: 0.209, *p*: 0.008). A positive correlation was detected between TRP and KNY as well as KYNA and 3HAA (r: 0.233, *p*: 0.003; r: 0.325, *p* < 0.001 and r: 0.238, *p*: 0.003, respectively).

**Figure 3:**
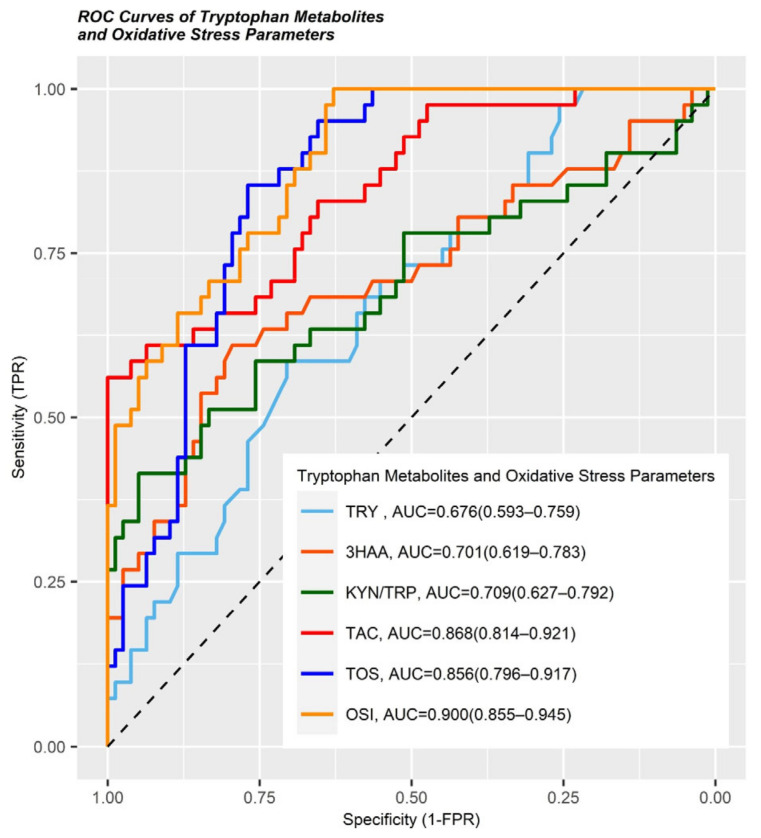
ROC curves of TRP, 3HAA, KYN/TRP, TAC, TOS, and OSI to distinguish IBD from healthy controls.

Table 3 and Figure 3 show that the AUCs of the serum TAC, TOS, and OSI for distinguishing IBD patients from healthy controls were 0.868, 0.856, and 0.9, respectively, while the AUCs of the TRP, 3HAA and KYN/TRP ratios for distinguishing IBD patients from healthy controls were 0.676, 0.701 and 0.709, respectively.

## 4. Discussion

Adequate consumption of TRP is essential for the maintenance of gut homeostasis. Evidence indicates that a tryptophan-deficient diet can disrupt the balance of gut microbiota and contribute to increased inflammation [13]. Moreover, Western-style eating habits can potentially have detrimental effects on the gut microbiome and metabolome, ultimately affecting intestinal immunity and contributing to the development and course of IBD [28]. Recent findings have demonstrated a significant connection between dietary therapy and clinical outcomes, particularly in relation to specific changes observed in compounds within the kynurenine and serotonin pathways. Ghiboub et al. reported that the levels of metabolites in the KP decrease early during clinical remission and remain reduced with ongoing dietary therapy and sustained remission, whereas there is an increase in specific compounds within the serotonin pathway as a result of sustained remission in pediatric CD [29]. Dietary intervention with TRP has been shown to exhibit an anti-inflammatory effect in experimental colitis models, regardless of nutritional status [30]. Endogenous TRP metabolites preserve intestinal immune-mediated homeostasis and microbial diversity in murine models, protecting against inflammation [3,31]. It was shown that mice deficient in TRP exhibited a worsening of colitis, greater reduction in weight, and decreased levels of intestinal antimicrobial peptides [32]. Tryptophan is essential for the in vivo synthesis of nicotinamide, also recognized as vitamin B3 or niacin, and inadequacy of niacin or tryptophan in the diet remains the underlying cause of pellagra, leading to the development of colitis in more than 90% of affected patients [32,33,34].

The catabolism of TRP to KYN by the rate-limiting enzyme IDO is closely controlled by proinflammatory molecules. Ferdinande et al. conducted an immunohistochemical analysis that revealed the upregulation of IDO in the colonic and ileal lesions of individuals diagnosed with UC and CD when compared to healthy individuals [35]. Another immunohistochemical analysis conducted on lesions obtained from 15 healthy subjects, 32 individuals with UC, and 19 cases with CD demonstrated that IDO was upregulated in patients with IBD, and its expression showed a positive correlation with the severity of the disease [36]. Sofia et al. reported that the serum KYNA/TRP ratio and the mucosal expression of IDO in patients with ulcerative colitis were positively correlated with endoscopic inflammation [21]. In a cohort study comprising 25 patients with CD and 10 healthy subjects, Gupta et al. reported that elevated KYN/TRP ratio and reduced TRP levels were observed in individuals with active CD and that these changes were significantly associated with alterations in inflammatory markers and the disease activity index [23]. It is conceivable that, as a result of increased IDO activity, serum TRP depletion results in increased conversion to KYN. In our study, we observed that the TRP concentration decreased and the KYN/TRP ratio increased in UC and CD patients, which may reflect raised IDO activity. Indeed, we noticed a trend indicating an increase in KYN levels of UC and CD in our study, but the effect did not reach statistical significance, possibly due to the limited number of enrolled patients and the high variability observed.

Concomitant assessment of TRP and its related compounds, including KYN as a direct precursor to KYNA, and 3HK as a second metabolite of KYN via an alternative metabolic pathway, showed a reduction in TRP and 3HAA levels without alterations in other metabolites across the entire group of UC and CD patients. These results concur with previous studies that have highlighted the insufficiency of TRP in individuals with IBD [23,37]. When we evaluated the ratio of metabolites to each other while KYN/TRP was reduced in the control group, the KYNA/KYN ratio was greater in the control group compared to UC and CD. However, there was no difference in the 3HK/KYN or 3HAA/3HK ratios between the groups.

In our study, we found that serum TAC levels decreased and serum TOS levels and OSI ratio increased in IBD patients, and these results may reflect that the balance between oxidant and antioxidant shifts in favor of oxidants in IBD patients. Yuksel et al. reported similar results, demonstrating a lower level of TAC in the IBD group, while TOS and OSI were greater than those in the control group [9]. In a study carried out by Sido et al. on individuals with CD, a raised oxidative stress level was observed in inflamed ileum mucosa compared to non-inflamed mucosa [38]. Szczeklik et al. showed that individuals with active CD had lower serum TAC levels than those with inactive CD and healthy controls [39]. At physiological pH, 3HAA functions as a free radical scavenger when metal ions are absent, but it behaves as a pro-oxidant in the presence of metal ions [40].

Several studies have indicated that TRP and its certain metabolites, including melatonin, KYNA, and xanthurenic acid, possess the ability to function as potent antioxidants in living organisms by eliminating reactive oxygen, reactive nitrogen, and active chlorine species, thereby enhancing the protection of the organism against oxidative damage caused by free radicals [41,42,43].

In clinical and research settings, determining appropriate cut-off points for continuous variables is important for decision-making and data interpretation. Various approaches for determining the optimal cut-off point have been suggested [44,45,46]. Although the Youden index, which we used to determine the cut-off in our study, is frequently used, it operates under the assumption that sensitivity and specificity are of equal importance. However, in clinical practice, this assumption may introduce bias, as the relative importance of sensitivity and specificity can vary depending on the clinical context. The Youden index may also be affected by the frequency and distribution of the dataset, particularly in imbalanced datasets. Due to the relatively balanced distribution of our dataset (78 controls and 80 IBD patients), the cut-off point selection remains unbiased. It should be noted that, depending on the method used to determine optimal cut-off, the cut-off value may vary based on the heterogeneity of the biomarker, potentially leading to inconsistency between cut-off points when interpreting the results.

Several limitations should be acknowledged, particularly the relatively small sample size and the single-center design in the Turkish population, which might reduce the broader applicability of the results. Since the control group did not undergo colonoscopy, the possibility of future development of IBD cannot be entirely ruled out. Another significant limitation is the lack of assessment of enzyme activities and related pathways, such as IDO activity and the serotonin pathway, which could have a substantial impact on the study’s outcomes and interpretations. Additionally, data on factors that could impact TRP levels and gut microbiota, such as patients’ dietary habits and antibiotic use within the past six months, are lacking. These limitations highlight the need for further research to address these gaps and validate the findings in more diverse and comprehensive settings.

## 5. Conclusions

In conclusion, the observation of impaired TRP and KP metabolites in individuals with IBD has prompted the hypothesis that KP could potentially contribute to the pathogenesis of IBD. Tryptophan and its metabolites may not only function as valuable biomarkers but may also hold promise as therapeutic targets in IBD.

## Figures and Tables

**Table 1:** Comparison of kynurenine pathway metabolite levels and routine laboratory parameters of patients with UC, CD, and control group.

	Control (*n* = 78)	CD (*n* = 41)	UC (*n* = 39)	*p*-Value
Demographic characteristics				
Age (years)	55.9 ± 9.2	56 ± 10.2	55.5 ± 8.4	0.971
Gender (Female/Male)	33 (42.3)/45 (57.7)	16 (39)/25 (61)	18 (46.2)/21 (53.8)	0.812
Glucose (mg/dL)	87.7 ± 19.4	90.4 ± 16.8	87.6 ± 16.3	0.714
Creatinine (mg/dL)	0.81 (0.74–0.94)	0.82 (0.74–0.96)	0.78 (0.69–0.92)	0.710
ALT (U/L)	17 (13–21.7)	19 (15–27)	17 (14–22.5)	0.066
AST (U/L)	16.5 (14–19.2)	17 (15–21)	16 (14.5–21)	0.605
WBC (K/uL)	6.84 (6.01–7.5)	7.51 (6.3–8.6)	7.55 (6.2–8.6)	0.06
RBC (K/uL)	5.37 (5.06–5.54)	5.45(5.19–5.58)	5.43 (5.13–5.58)	0.572
HCT (%)	48.8 ± 3.23	49.7 ± 2.40	49.9 ± 2.19	0.080
MCV (fL)	92.3 (88.4–94.4)	92.9 (90.7–94.6)	92.5 (90.3–95.9)	0.479
PLT (K/uL)	229 (195–260)	221 (197–249)	235 (205–253)	0.825
Tryptophan metabolites				
Tryptophan (ng/mL)	25,100 (19,625–32,475) ^a^	19,700 (17,100–25,800) ^b^	19,900 (17,550–24,600) ^b^	<0.001
Kynurenine(ng/mL)	41.2 (31.4–57.3)	52.5 (34.8–58.9)	49.4 (34.9–67.7)	0.265
Kynurenic acid (ng/mL)	0.74 (0.5–0.96)	0.61 (0.44–0.76)	0.61 (0.47–0.84)	0.072
3HK (ng/mL)	14 (9.5–18.4)	11.1 (7–17.5)	14.6 (8.5–19.3)	0.309
3HAA (ng/mL)	38.2 (29.5–58.5) ^a^	24.8 (15.9–41.7) ^b^	27.2 (15.5–36.2) ^b^	<0.001
KYN/TRP	1.6 (1.3–2.1) ^a^	2.2 (1.6–3.2) ^b^	2.5 (1.6–3.5) ^b^	<0.001
KYNA/KYN	15.7 (10.7–23) ^a^	12.9 (9.4–15.9) ^b^	12.4 (8.8–17) ^b^	0.006
3HK/KYN	28.9 (20.6–51.9)	25.1 (14.5–41.2)	28.8 (16.9–39.4)	0.211
3HAA/3HK	2.86 (1.87–5.23)	2.3 (0.91–4.37)	2.35 (1.08–4.74)	0.127
Oxidative stress parameters				
TOS (µM H_2_O_2_ Eq)	10.35 (9.18–11.81) ^a^	13.27 (12.29–14.41) ^b^	13.30 (12.42–14.77) ^b^	<0.001
TAC (mMAskorbate Eq)	1.06 ± 0.14 ^a^	0.83 ± 0.16 ^b^	0.82 ± 0.13 ^b^	<0.001
OSI	9.66 (8.31–12.33) ^a^	16.31 (12.86–22.02) ^b^	16.01 (13.75–20.48) ^b^	<0.001

Data were presented as mean ± standard deviation or median with interquartiles (25th percentile–75th percentile) for numerical variables and were described as the count (*n*) and percentage (%) for categorical variables. Different letters represent statistically significant differences.

**Table 2:** Correlation analysis of laboratory parameters.

		TRP	KYN	KYNA	3HK	3HAA	TOS	TAC
TRP	*r*	1.000	0.233 *	0.325 *	−0.068	0.238 *	0.064	−0.097
	*p*	.	0.003	<0.001	0.397	0.003	0.424	0.223
KYN	*r*	0.233 *	1.000	0.367 *	0.039	0.097	−0.194 *	0.046
	*p*	0.003	.	0.000	0.625	0.227	0.015	0.568
KYNA	*r*	0.325 *	0.367 *	1.000	−0.063	0.225 *	−0.092	0.190 *
	*p*	<0.001	<0.001	.	0.429	0.004	0.249	0.017
3HK	*r*	−0.068	0.039	−0.063	1.000	−0.106	−0.276 *	0.233 *
	*p*	0.397	0.625	0.429	.	0.186	<0.001	0.003
3HAA	*r*	0.238 *	0.097	0.225 *	−0.106	1.000	0.209 *	−0.191 *
	*p*	0.003	0.227	0.004	0.186	.	0.008	0.016
TOS	*r*	0.064	−0.194 *	−0.092	−0.276 *	0.209 *	1.000	−0.666 *
	*p*	0.424	0.015	0.249	<0.001	0.008	.	<0.001
TAC	*r*	−0.097	0.046	0.190 *	0.233 *	−0.191 *	−0.666	1.000
	*p*	0.223	0.568	0.017	0.003	0.016	0.000	.

* indicates statistically significant differences.

**Table 3:** ROC curve analysis and statistical diagnostic measures of laboratory parameters.

	AUC	Cut-off Point	Sensitivity (%)	Specificity (%)	PPV (%)	NPV (%)
TRP	0.676 (0.593–0.759)	<25,000	76.2	51.3	61.6	67.8
3HAA	0.701 (0.619–0.783)	<27.3	56.2	80.8	75	64.3
KYN/TRP	0.709 (0.627–0.792)	≥2.676	46.3	93.6	88.1	62.9
TOS	0.856 (0.796–0.917)	≥11.87	85	76.9	79.1	83.3
TAC	0.868 (0.814–0.921)	<0.86	61.3	93.6	90.7	70.2
OSI	0.900 (0.855–0.945)	≥11.2	98.8	64.1	73.8	98

## Data Availability

Data can be shared on reasonable request.
